# Peripheral Parking: A Bailout Strategy for Managing Dislodged Coronary Stents

**DOI:** 10.7759/cureus.88757

**Published:** 2025-07-25

**Authors:** Satyajit Padhiary, Devesh Kumar, Anbhigya Kumar Arya, Maaz Ahmad Khan, Saurabh Kumar Singh

**Affiliations:** 1 Cardiology, Vardhman Mahavir Medical College and Safdarjung Hospital, New Delhi, IND

**Keywords:** acute coronary syndrome, bailout technique, interventional cardiology complications, peripheral parking, stent embolization

## Abstract

Coronary stent dislodgement is a rare but potentially life-threatening complication during percutaneous coronary intervention (PCI). It may occur due to complex anatomy, heavy calcification, tortuous vessels, or equipment malfunction, leading to embolization, vessel injury, or myocardial infarction if not promptly addressed. We present a case of stent dislodgement during transradial PCI, wherein a dislodged undeployed coronary stent migrated to the peripheral arterial system (left common iliac artery) seen on non-contrast computed tomography (NCCT). On subsequent fluoroscopy, the stent was seen aligned with the vessel contour and with good flow across it, so parked permanently without compromising distal flow or limb perfusion. The patient remained hemodynamically stable, and no short-term vascular complications were observed. This case highlights the peripheral parking technique as a practical bailout strategy in scenarios where retrieval is not feasible and the stent is inaccessible or poses a risk of further embolization.

## Introduction

Coronary stents are now a cornerstone of percutaneous coronary intervention (PCI), significantly enhancing patient outcomes. However, complications such as stent dislodgement or embolization, although rare, remain clinically significant. Stent embolization refers to the unintended displacement of the stent after partial or failed deployment. This can result in serious complications, including coronary artery thrombosis, acute myocardial infarction, or sudden cardiac death. Moreover, if the stent migrates beyond the coronary vasculature, it may lead to systemic embolization, increasing the risk of cerebrovascular events or peripheral arterial occlusion. Early recognition and prompt management are critical to minimizing these risks [1]. Several factors can contribute to stent dislodgement, including coronary calcification, tortuous vessel anatomy, diffuse fibrotic disease, and the presence of previously implanted stents [2].

In addition, the stent’s design, such as strut thickness and metal alloy composition, plays a crucial role. Despite significant advances in stent technology, improved lesion preparation techniques, and widespread adoption of drug-eluting stents (DES), the incidence of stent dislodgement remains approximately 1% [3].

Several catheter-based strategies can be employed to recover a dislodged coronary stent; the selection depends on the stent's position, the vessel size, and the complexity. These strategies include balloon anchoring, snare retrieval, double-wire knotting or braiding, basket or fragment retrievers, the crush-and-stent technique, and, last but not least, the peripheral parking technique [4].

Here, we present a complex case of complete stent dislodgement during PCI of the left main coronary to left anterior descending (LM-LAD) artery, followed by embolization to the left common iliac artery, managed with the peripheral parking technique.

## Case presentation

**Figure 1:**
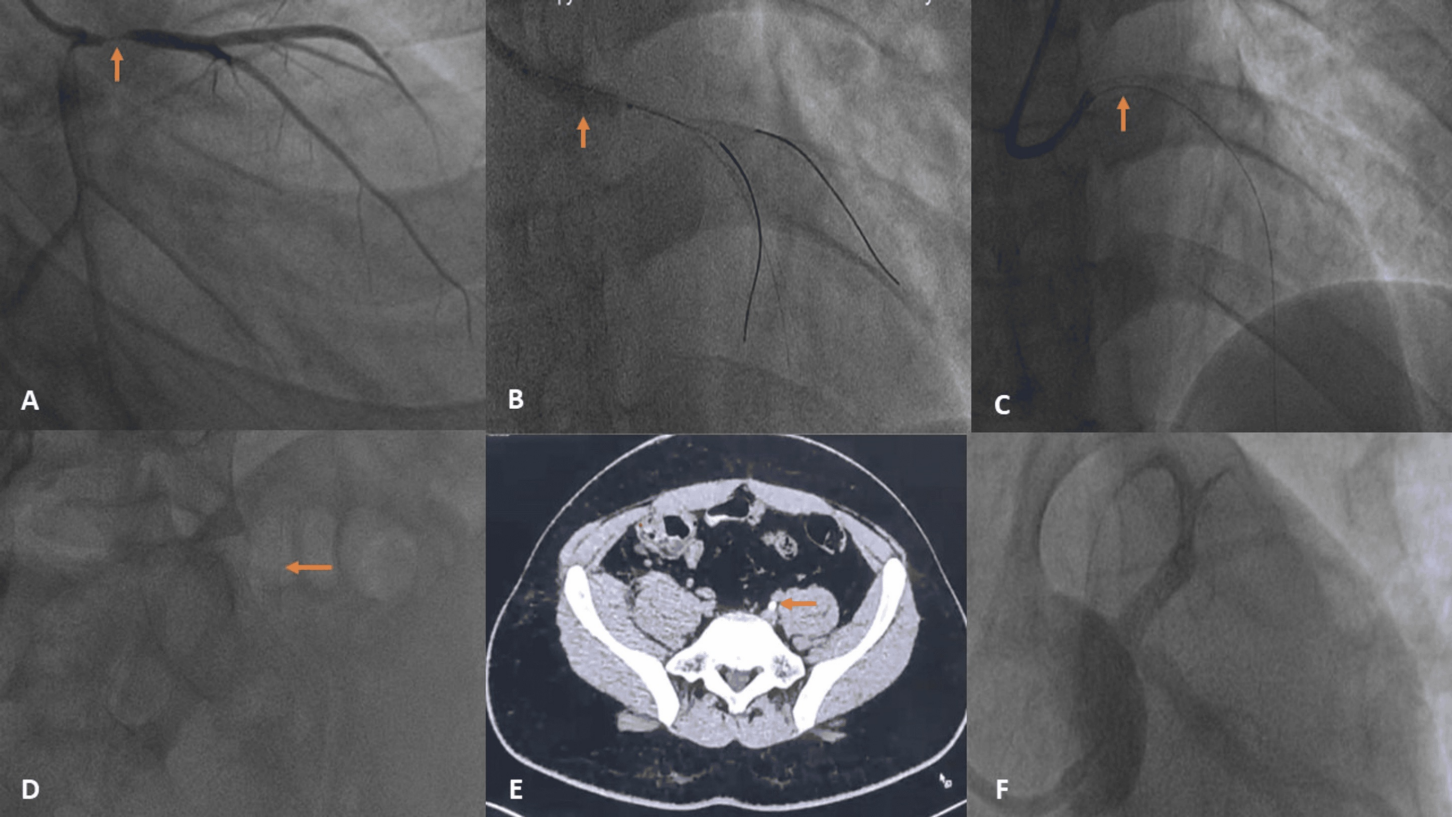
Coronary angiograms, fluoroscopy, and NCCT images. (A) Coronary angiogram of the left coronary system taken in the right anterior oblique (RAO) caudal view, which reveals a tubular lesion (arrow) in the ostio-proximal left anterior descending (LAD) artery extending into the distal left main coronary artery. (B) Coronary angiogram in the left anterior oblique cranial view with stent being deployed from left main ostia to the ostio-proximal LAD (arrow). (C) The result post-deployment of the stent in left anterior oblique (LAO) cranial view (arrow). (D) The left main stent has embolized to the left common iliac artery as seen on fluoroscopy (arrow). (E) The stent (arrow) in a cross-sectional view on NCCT of the abdomen and pelvis. Part F reveals the final result of the procedure after deploying the second stent in the left main coronary artery in the LAO caudal view with TIMI 3 flow. NCCT: non-contrast computed tomography; TIMI: thrombolysis in myocardial infarction

A 30-year-old male without prior comorbidities presented to the emergency room with complaints of typical chest pain for 8 hours prior to admission. At presentation, pulse rate was 89 beats/minute, blood pressure was 110/80 mmHg, and respiratory rate was 14 breaths/minute. A 12-lead electrocardiogram (EKG) showed sinus rhythm with ST segment elevation in V1-4 suggestive of anterior wall myocardial infarction (Figures 1A-1F). Serum troponin I levels were elevated at 10.2 ng/mL. Transthoracic echocardiogram revealed anterior wall hypokinesia with a left ventricular ejection fraction of 45%. The patient underwent coronary angiogram via right radial access, which showed a tubular lesion from distal left main to proximal LAD with maximum stenosis of 95% (Figures 1A-1F).

A 6 French Judkins left guiding catheter (JL 3.0) (Minneapolis, MN: Medtronic) was engaged. The lesion was successfully crossed with a workhorse wire (run-through) (Tokyo, Japan: Terumo). The lesion was dilated using a 2×15 mm semi-compliant balloon at the site of the maximum stenosis. After lesion preparation, a 3.5×18 mm Everolimus-eluting stent (XIENCE Xpedition; Chicago, IL: Abbott Vascular) was implanted (Figures 1B, 1C). Post-dilatation of the stent was performed using a 4.5×8 mm non-compliant Pantera Leo balloon (Berlin, Germany: Biotronik), from the distal to the ostial LM. The final angiogram revealed TIMI 3 flow across LM-LAD; however, the stent was not visible. As our patient was hemodynamically stable, we explored all possible avenues, including removing the entire system, to locate the displaced stent, but we were unable to find it. So, we decided to predilate the lesion with a bigger SC balloon of 2×15 mm size. This time, a 3.5×28 mm Xience Xpedition stent was deployed, and post-dilation was performed with a 4.5×8 mm NC balloon (Figure 1F). We carried out a non-contrast computed tomography (NCCT) scan to identify the displaced stent, which was located in the left common iliac artery (Figure 1E). On subsequent fluoroscopy, the stent was seen aligned with the vessel contour and with good flow across it (Figure 1D). Doppler ultrasound performed on the third day of the hospital stay indicated normal triphasic flow in the common femoral artery and distal areas. The patient had an uneventful course and was subsequently discharged after three days. The patient is doing well at one month of follow-up.

## Discussion

Stent dislodgement during percutaneous coronary intervention (PCI) is an uncommon but potentially life-threatening complication, with an incidence ranging from 0.32% to 3.4%. [2] Over the past two decades, its frequency has declined significantly due to advancements in stent technology, including the use of pre-mounted stents, improved cross-sectional profiles, enhanced delivery systems, and growing operator expertise [5,6].

Several clinical and anatomical scenarios increase the risk of stent embolization. These include severely calcified lesions, significant vessel angulation, direct stenting in critical lesions, manual manipulation of the stent system, previously deployed proximal stents, and inadequate guidewire or catheter support [6]. Meticulous lesion preparation, especially with pre-dilatation, substantially reduces the risk of stent loss.

Various techniques have been described for managing embolized stents. These include crushing the embolized stent against the vessel wall using a balloon or another stent; retrieving it using a snare device; passing a second wire beyond the lost stent and twisting it with the primary wire to engage the stent struts before removal; and, as in our case, advancing a small balloon distal to the embolized stent, inflating it, and withdrawing the entire assembly [7]. The "peripheral parking technique" is a novel approach in which the embolized stent is intentionally deployed in a peripheral vessel when retrieval is deemed unsafe or impractical [8].

Stent loss can result in serious complications such as acute myocardial infarction, coronary thrombosis, cerebrovascular embolic events (especially if embolized into the ascending aorta), and may necessitate emergency coronary artery bypass grafting (CABG) [9]. In some instances, it can be fatal. A study by Eggebrecht et al. reported a 15% mortality rate among patients with failed stent retrieval, while Alfonso et al. found no adverse outcomes in nine patients with retained stents [9-11].

In the present case, stent embolization was likely precipitated by suboptimal lesion preparation (inadequate pre-dilatation), selection of a shorter stent, and improper deployment. The absence of intravascular ultrasound (IVUS) guidance may have contributed to insufficient procedural precision [12]. The dislodged stent migrated to the left common iliac artery but remained asymptomatic, with no evidence of thrombosis, ischemia, or vessel wall injury.

To prevent such complications, we recommend comprehensive lesion preparation, including pre-dilatation; use of adjunctive techniques such as rotational or orbital atherectomy or intravascular lithotripsy, when indicated; selection of appropriately sized stents; ensuring adequate guide catheter support; and minimizing excessive manipulation. Adequate sedation should also be ensured to optimize patient cooperation and procedural success.

## Conclusions

Stent embolization, although a rare complication during PCI, poses significant clinical challenges and potential life-threatening risks. The peripheral parking technique offers a pragmatic and safe solution when conventional retrieval methods fail or are deemed too risky. By guiding the dislodged stent to a non-critical peripheral vascular bed, such as the iliac or femoral artery, this approach helps avoid urgent surgical intervention and reduces immediate complications. However, careful patient selection, vigilant follow-up, and long-term monitoring for vascular sequelae are essential to ensure optimal outcomes. This technique exemplifies how strategic innovation in interventional cardiology can mitigate procedural risks and enhance patient safety.

Although stent embolization during PCI is a rare occurrence, operators must be aware of this possibility and the potential strategies for managing such a situation. In some cases, leaving the stent in place is the preferable choice over intervening, as demonstrated in our situation.
